# Exposure to Bisphenol A Prenatally or in Adulthood Promotes T_H_2 Cytokine Production Associated with Reduction of CD4^+^CD25^+^ Regulatory T Cells

**DOI:** 10.1289/ehp.10829

**Published:** 2008-01-29

**Authors:** Huimin Yan, Masaya Takamoto, Kazuo Sugane

**Affiliations:** Department of Infection and Host Defense, Division of Immunology and Infectious Diseases, Shinshu University Graduate School of Medicine, Matsumoto, Japan

**Keywords:** bisphenol A, cytokine, endocrine-disrupting chemicals, prenatal exposure, regulatory T-cells

## Abstract

**Background:**

Bisphenol A (BPA) is a widespread endocrine-disrupting chemical that can affect humans and animals.

**Objectives:**

We investigated the effects of adult or prenatal exposure to BPA on T-helper (T_H_)1/T_H_2 immune responses and the mechanisms underlying these effects.

**Methods:**

To evaluate the effects of exposure to BPA in adulthood, male *Leishmania major*–susceptible BALB/c and –resistant C57BL/6 mice were subcutaneously injected with 0.625, 1.25, 2.5, and 5 μmol BPA 1 week before being infected with *L. major*. To evaluate prenatal exposure, female mice were given BPA-containing drinking water at concentrations of 1, 10, and 100 nM for 2 weeks, then mated, and given BPA for another week. Male 10-week-old offspring were infected with *L. major*. Footpad swelling was assessed as a measure of the course of infection.

**Results:**

Mice exposed to BPA prenatally or in adulthood showed a dose-dependent increase in footpad swelling after being infected with *L. major*. Exposure to BPA in adulthood significantly promoted antigen-stimulated production of interleukin (IL)-4, IL-10, and IL-13 but not interferon-γ (IFN-γ). However, mice prenatally exposed to BPA showed increased production of not only IL-4 but also IFN-γ. The percentages of CD4^+^CD25^+^ cells were decreased in mice exposed to BPA either prenatally or in adulthood. Effects of prenatal BPA exposure were far more pronounced than effects of exposure in adulthood.

**Conclusion:**

BPA promotes the development of T_H_2 cells in adulthood and both T_H_1 and T_H_2 cells in prenatal stages by reducing the number of regulatory T cells.

Bisphenol A (BPA), an estrogenic endocrine-disrupting chemical (EDC), has drawn attention because of its potential for human exposure. BPA is widely used, including in dentistry, food packaging, and lacquers to coat food cans and water pipes. It can enter the environment, resulting in chronic exposure of humans and wildlife. In fact, BPA has been detected not only in food and water (Heemken et al. 2001; Shin et al. 2001; Thomson and Grounds 2005) but also in human urine and blood samples as well as in the placenta and amniotic fluid of pregnant women (Ikezuki et al. 2002; Matsumoto et al. 2003; Schonfelder et al. 2002; vom Saal and Hughes 2005). BPA is one of the most widespread EDCs.

There is much evidence that exposure to BPA through contamination of the environment or the treatment of experimental animals disrupts developmental programs to alter sexual phenotypes and reproductive functions (Farabollini et al. 2002; Herath et al. 2004). BPA antagonizes the actions of thyroid hormone (Moriyama et al. 2002). Exposure of pregnant rats to BPA resulted in the chemical’s transplacental transfer to the fetuses (Takahashi and Oishi 2000; Welshons et al. 2006), suggesting that developing embryos or fetuses might be affected by BPA. Prenatal exposure to BPA has been shown to alter a variety of reproductive endocrine parameters, such as testosterone and luteinizing hormone levels in rats (Ramos et al. 2003; Rubin et al. 2001) and the early onset of sexual maturation of female mice (Honma et al. 2002). In addition, behavioral changes have been reported in offspring of mice exposed to BPA during pregnancy and lactation (Dessi-Fulgheri et al. 2002). BPA may also be a potentially important modulator of immune responses. It inhibits adhesion capacity and promotes cytokine production in macrophages *in vitro* (Segura et al. 1999; Yamashita et al. 2005). Exposure to BPA also enhances the production of autoantibodies by B1 cells (Yurino et al. 2004). Furthermore, imbalanced T-helper (T_H_)1/T_H_2 immune responses have been demonstrated on exposure to BPA. BPA inhibits the secretion of interferon-γ (IFN-γ) in C57BL/6 and female NZB/NZW mice (Sawai et al. 2003). In contrast, BALB/c mice treated with BPA exhibit augmented T_H_1 immune responses alone (Alizadeh et al. 2006), or both T_H_1 and T_H_2 responses (Yoshino et al. 2003). Our previous study indicated that BPA promotes T_H_2 cytokine production *in vitro* and *in vivo* (Tian et al. 2003). However, the effects of prenatal exposure to BPA on immune responses have not been clarified.

In this study, we used mice infected cutaneously with *Leishmania major* to investigate the effect of BPA on T_H_1/T_H_2 immune responses in adulthood and prenatal stages. The model provides an excellent system with which to study the factors controlling the generation and regulation of T_H_1 and T_H_2 cells *in vivo*. Experimental infections of different strains of mice with *L. major* result in the development of either a predominant T_H_1 response and resistance or a predominant T_H_2 response and susceptibility. The early production of interleukin-12 (IL-12) and IFN-γ promotes a T_H_1 response and healing, whereas IL-4 production is necessary for the development of a T_H_ 2 response and of progressive disease. We also focused on CD4^+^CD25^+^ regulatory T cells (Treg cells), one of the CD4^+^ T cell populations constitutively expressing the IL-2 receptor α-chain (CD25) playing a central and prominent role in the maintenance of the immunologic balance (Maloy and Powrie 2001; Shevach 2002) by inhibiting the proliferation of and the production of cytokines by CD4^+^ and CD8^+^ T cells (Dieckmann et al. 2005; Stassen et al. 2004). We evaluated whether CD4^+^CD25^+^ Treg cells were affected by exposure to BPA, resulting in the alteration of cytokine production by CD4^+^ T cells.

## Materials and Methods

### Mice

Six- to 8-week-old *L. major*–susceptible BALB/c and *L. major*–resistant C57BL/6 mice were purchased from Clea Japan (Tokyo, Japan). Mice were housed in polymethylpentene (TPX) cages and fed sterile standard chow (FR-2; Funabashi Farm, Chiba, Japan). Drinking water was provided *ad libitum* in glass bottles. All animals were handled according to the guidelines of the Ethics Committee for Animal Experiments of Shinshu University. Animals were treated humanely and with regard for alleviation of suffering.

### Monoclonal antibodies and reagents

BPA was purchased from Nacalai Tesque (Kyoto, Japan). Phycoerythrin (PE)-conjugated anti-CD4 and fluorescein isothiocyanate (FITC)–conjugated anti-CD25 monoclonal antibodies (mAbs) were obtained from BD, Biosciences (San Diego, CA, USA). The cytometric bead array (CBA) kits were also from BD Biosciences.

### Leishmania major

*L. major* (MHOM/SU/73/5ASKH) was kept in a virulent state by continuous passage in BALB/c mice. A cell suspension of popliteal lymph node from an infected BALB/c mouse was cultured in Schneider’s medium (Gibco BRL, Gaithersburg, MD, USA) supplemented with 20% heat-inactivated fetal calf serum (FCS; Biocell Laboratories, Carson, CA, USA). Stationary phase promastigotes were collected by centrifugation and washed with saline. Mice were infected in the right hind footpad with 5 × 10^6^ promastigotes. The course of infection was monitored by making weekly measurements of footpad thickness with a metric caliper. The results were expressed as the difference between the thickness of the infected right footpad and that of the noninfected left one.

To prepare soluble *L. major* antigen, 1 × 10^9^ promastigotes were homogenized by three cycles of freezing and thawing in phosphate-buffered saline. Aliquots were stored at −30°C before use.

### BPA treatment

#### Exposure of adult male mice to BPA

BPA was dissolved in corn oil and injected subcutaneously into the right hind leg at doses of 0.625, 1.25, 2.5 and 5 μmol, which is equivalent to 5.7, 11.4, 22.8, and 45.6 mg/kg body weight (bw). These doses were based on our previous study in which 1 μmol BPA was shown to increase IL-4 and IL-10 production in *Trichinella spiralis*–infected mice (Tian et al. 2003). The control mice received corn oil vehicle alone. One week later, the mice were injected with *L. major* promastigotes in the footpad of the same leg.

#### Prenatal exposure to BPA

Female mice were given BPA in drinking water at doses of 1, 10, and 100 nM for 2 weeks. Each group of mice was then mated with a male and treated with BPA-containing drinking water for another week. Offspring born within 16–19 days after BPA treatment was complete were used in this experiment. The 100 nM (about 3 μg/kg bw/day) dose of BPA was based on recent studies showing that administration of low doses of BPA at 2 and 20 μg/kg bw/day to pregnant animals caused permanent changes in reproductive organs of offspring (Honma et al. 2002; Nagel et al. 1997). The mice in all groups drank approximately 3–4 mL water per day. The total dose received by each female mouse during the period of experiment was about 0.07, 0.7, or 7 nmol. Offspring of dams who received drinking water without BPA were used as controls. Male 10-week-old offspring were infected with *L. major*.

### In vitro *culture of splenocytes*

A single-cell suspension containing 2 × 10^6^ splenocytes from each mouse was incubated in 24-well tissue-culture plates (Greiner, Nurtingen, Germany) in 1 mL RPMI 1640 medium (Nissui Pharmaceutical Co., Tokyo, Japan) supplemented with 10% FCS (Biocell Laboratories), penicillin (100 IU/mL), and streptomycin (100 μg/mL) (Gibco BRL) at 37°C in a humidified atmosphere of 5% CO_2_ and 95% air. Cells were stimulated with *L. major* antigen (3 μg/mL) during the cultivation. Culture supernatants were collected 48 hr later and stored frozen until used.

### Cytokine analysis

Concentrations of IL-4, IL-10, IL-13, and IFN-γ in culture supernatants were determined using CBA kits according to the manufacturer’s instructions.

### Flow cytometric analysis

Single-cell suspensions containing 1 × 10^6^ splenocytes were stained with PE-conjugated anti-CD4 mAb and FITC-conjugated anti-CD25 mAb. The cells were washed, then analyzed using fluorescence-activated cell sorting (FACS) with a FACSCalibur flow cytometer (BD Biosciences) with CellQuest software (BD Biosciences).

### Statistical analysis

Results are presented as the mean ± SE. The statistical significance of the values was evaluated using Student’s *t*-test. The significance was assessed at the *p* < 0.05 level of confidence.

## Results

### Effects of BPA on footpad swelling and cytokine production in L. major–infected adult male mice

**Figure 1:**
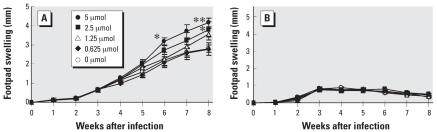
Effects of exposure to BPA in adult male BALB/c (*A*) and C57BL/6 (*B*) mice on footpad swelling after infection with *L. major*. Values represent mean ± SE (*n* = 3–4). *p < 0.05 and **p < 0.01 compared with the nonexposed control group.

Adult male mice injected with different doses of BPA were infected with promastigotes of *L. major* 1 week later. *L. major*–susceptible BALB/c mice developed a continuous increase in footpad thickness whether or not they were injected with BPA. The degree of swelling increased dose-dependently in mice treated with BPA. Mice exposed to 2.5 and 5 μmol of BPA developed significantly larger swelling than nonexposed control mice at weeks 6 and 8 after infection. Eight weeks after infection, footpad swelling was 1.49-fold greater in mice treated with 5 μmol of BPA than in controls (Figure 1A). However, infection with *L. major* among resistant C57BL/6 mice resulted in minimal swelling that began to resolve by 4 weeks after infection. There was no significant difference in footpad swelling among the groups (Figure 1B).

**Figure 2:**
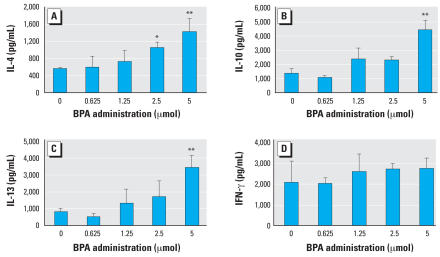
Effects of exposure to BPA in adult male BALB/c mice on IL-4 (*A*), IL-10 (*B*), IL-13 (*C*), and IFN-γ (*D*) cytokine production after infection with *L. major*. Values represent mean ± SE (*n* = 3–4). *p < 0.05 and **p < 0.01 compared with the nonexposed control group.

The administration of BPA resulted in an increase in the production of IL-4 by *L. major* antigen–stimulated splenocytes from *L. major*–infected BALB/c mice at week 8 in a dose-dependent manner. IL-4 levels were significantly higher in mice treated with 2.5 and 5 μmol of BPA than in untreated control mice. In addition, augmented production of IL-10 and IL-13 was observed in mice exposed to 5 μmol of BPA. However, no significant differences in levels of IFN-γ were observed between the untreated and BPA-treated groups (Figure 2). No significant differences in levels of production of T_H_1/T_H_2 cytokines were observed between untreated and BPA-treated C57BL/6 mice (data not shown).

### Change in the percentage of CD4^+^CD25^+^ T cell in BPA-treated adult male mice

**Figure 3:**
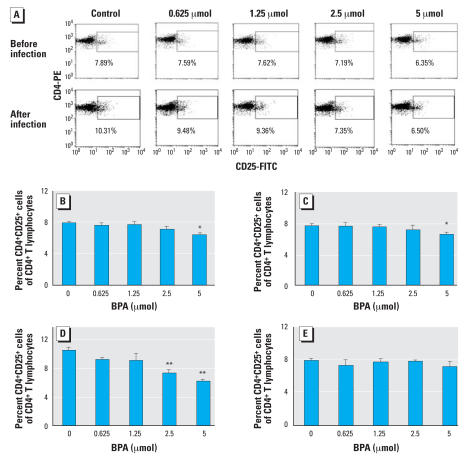
Effects of exposure to BPA on the percentage of CD4^+^CD25^+^ cells among CD4^+^ T cells in adult male mice. (*A*) The representative FACS data of BALB/c mice before and after infection with *L. major*. (*B–E*) Splenocytes from BALB/c (*B*, *D*) and C57BL/6 (*C*, *E*) mice before (*B*, *C*) and 8 weeks after (*D*, *E*) infection. Values represent mean ± SE (*n* = 3–4). *p < 0.05 and **p < 0.01 compared with the nonexposed control group.

The percentages of CD4^+^CD25^+^ cells among CD4^+^ T cells decreased significantly 1 week after treatment with 5 μmol of BPA in both BALB/c and C57BL/6 mice. Eight weeks after *L. major* infection, increased percentages of CD4^+^CD25^+^ cells were found in nonexposed susceptible BALB/c but not in resistant C57BL/6 mice. The percentages of CD4^+^CD25^+^ cells were significantly lower in BALB/c mice exposed to BPA at 2.5 and 5 μmol than in nonexposed mice. In contrast, no significant differences were seen between BPA-treated and nontreated C57BL/6 mice (Figure 3).

### Effects of prenatal exposure to BPA on footpad swelling and production of IL-4 and IFN-γ in L. major–infected male offspring

**Figure 4:**
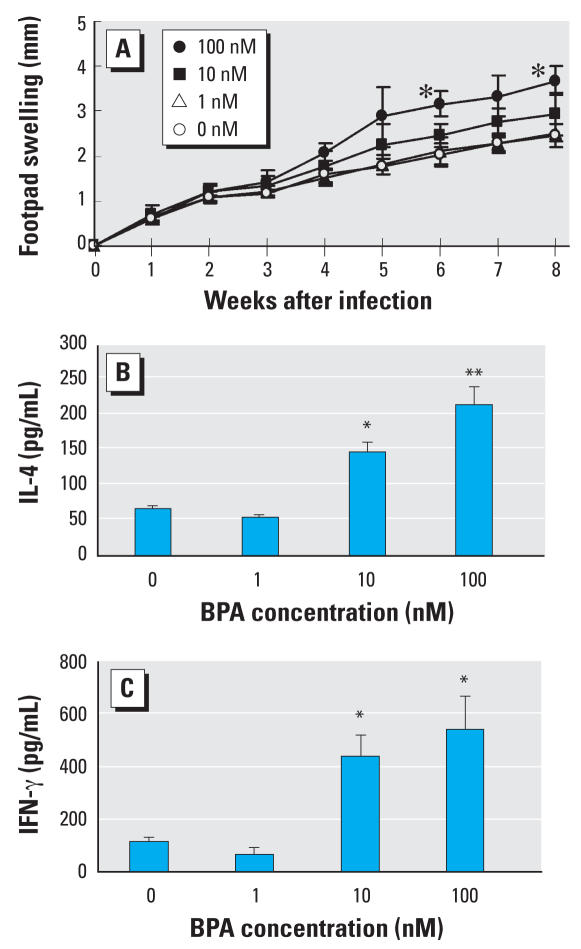
Effects of prenatal exposure to BPA on the course of *L. major* infection and T_H_1- and T_H_2- related cytokine production. (*A*) Footpad swelling after infection with *L. major*. (*B, C*) Splenocytes were obtained at 8 weeks after infection and cultured for 48 hr with *L. major* antigen. Concentrations of IL-4 (*B*) and IFN-γ (*C*) in culture supernatants were determined using CBA kits. Values represent the mean ± SE (*n* = 4). *p < 0.05 and **p < 0.01 compared with the control group.

Female BALB/c mice were given drinking water containing 1, 10, or 100 nM BPA for 2 weeks. They were then mated with male mice and given BPA-containing drinking water for another week. Male offspring were challenged with 5 × 10^6^ stationary-phase promastigotes of *L. major* in the hind footpad at week 10 after birth. The footpad swelling increased rapidly in the nonexposed as well as all the BPA-exposed groups (Figure 4A). Offspring of mice exposed to 100 nM BPA developed significantly larger swelling than controls at weeks 6 and 8 after infection. Eight weeks after infection, footpad swelling was 1.50-fold larger in offspring born to dams exposed to 100 nM BPA than in controls .

Production of IL-4 by splenocytes was significantly increased in offspring from dams exposed to 10 and 100 nM BPA but not in those born to 1 nM BPA-treated females compared with the nonexposed control mice. Similar results were observed in IFN-γ production. Mice showing increased footpad swelling demonstrated increased production of both T_H_1 and T_H_2 cytokines (Figure 4B,C).

### Change in the percentage of CD4^+^CD25^+^ T cell in male mice exposed prenatally to BPA

**Figure 5:**
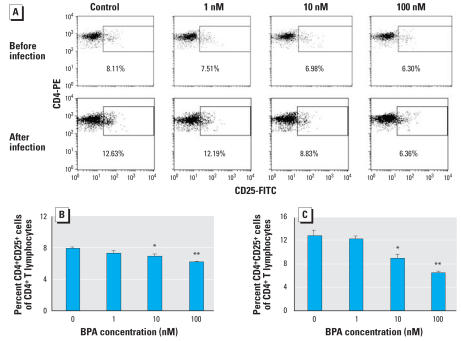
Effects of prenatal exposure to BPA on the percentage of CD4^+^CD25^+^ cells among CD4^+^ T cells. (*A*) The representative FACS data of offspring on day 22 of birth (before infection) and after *L. major* infection. (*B, C*) Splenocytes from offspring of dams exposed to the indicated dose of BPA were obtained on day 22 of birth (*B*) and 8 weeks after *L. major* infection (*C*), and stained with PE-conjugated anti-CD4 and FITC-conjugated anti-CD25 mAbs. CD4^+^ lymphocytes were gated and percentages of CD25^+^ cells were determined. Values represent mean ± SE (*n* = 3–4). *p < 0.05 and **p < 0.01 compared with the nonexposed control group.

Before infection, a dose-dependent decrease in the percentages of CD4^+^CD25^+^ cells among CD4^+^ T cells was observed in offspring of dams exposed to BPA. The percentages of CD4^+^CD25^+^ cells increased significantly after infection with *L. major*. The difference in the percentage of CD4^+^CD25^+^ cells became larger between offspring born to dams exposed to BPA and nonexposed mice (Figure 5).

## Discussion

In the present article, we clearly demonstrate the effects of exposure to BPA on immune responses using mice infected with *L. major*. Mice exposed to BPA prenatally or in adulthood showed a dose-dependent increase in footpad swelling after being infected with *L. major*. BPA promoted the production of IL-4 and other cytokines in each case. Similar results were seen in adult mice infected with a nematode, *T. spiralis* (Tian et al. 2003). Especially, a smaller amount of BPA could affect the immune responses of the next generation. Promotion of cytokine production was associated with decreases in CD4^+^CD25^+^ Treg cells, indicating that BPA exerted its effects by reducing the number of Treg cells.

Exposure to BPA by subcutaneous injection in adulthood significantly promoted antigen-stimulated production of IL-4, IL-10, and IL-13 in T_H_2-skewed BALB/c mice infected with *L. major*. However, oral administration with BPA resulted in an insignificant increase of T_H_2 cytokine production and footpad swelling after infection with *L. major* (data not shown). Subcutaneous injection in the leg with BPA more effectively altered immune responses after *L. major* infection in the footpad than did oral administration.

BPA exposure comes from multiple sources. Although oral delivery appears to be most relevant for extrapolation to humans, other delivery routes may reveal effects of BPA (Richter et al. 2007). BPA has been reported to leach from hemodialyzers into the serum (Haishima et al. 2001). The concentration of BPA was much higher in sera of dialysis patients than in those of healthy subjects (Murakami et al. 2007). Our observation gives a warning that immune responses in these patients would be affected by BPA.

In contrast to BALB/c mice, T_H_1-skewed C57BL/6 mice showed no significant increase in cytokine production and footpad swelling, suggesting that exposure to BPA in adult mice did not influence T_H_1 cells. This is consistent with the observation that BPA promotes *in vitro* IL-4 production by T_H_2 cells from *L. major*–infected BALB/c mice, but not IFN-γ production by T_H_1 cells from C57BL/6 and BALB/c mice (Tian et al. 2003). In resistant C57BL/6 mice, low levels of IL-4 are produced only transiently (Launois et al. 1997). Therefore, IL-4 production could not be promoted by BPA. These results indicate that BPA promotes T_H_2 cytokine production but not a change in the balance of immune responses from T_H_1 toward T_H_2. Other EDCs, such as tributyltin and *p*-*n*-nonylphenol, have also been reported to induce T_H_2 polarization (Iwata et al. 2004; Kato et al. 2004). T_H_2 immune response might be easily enhanced by EDCs.

The effects of BPA on the developing immune system in embryos or fetuses have not been elucidated. BPA can leak from the placenta and accumulate in the fetus (Miyakoda et al. 1999; Takahashi and Oishi 2000; Zalko et al. 2003). Additionally, there is increasing evidence that the development of the fetal immune system is regulated by the maternal immune system (Warner 2004). BPA influences the immune responses in adult mice; it is therefore possible that maternal exposure to BPA may affect the immune function of the next generation. In this study, we investigated whether exposure to low doses of BPA during the early periods of immune development could induce immunotoxic effects. We showed that prenatal exposure to BPA increased the production of a T_H_1 cytokine, IFN-γ, and a T_H_2 cytokine, IL-4, after the offspring developed, suggesting that prenatal exposure to BPA can induce persistent immunologic effects lasting into adulthood. These results are consistent with a previous report that fetal exposure to BPA augmented T_H_1 and T_H_2 immune responses (Yoshino et al. 2004). Although prenatal exposure to BPA led to increased IFN-γ production, these offspring failed to control disease progression following challenge with *L. major*. This may be due to the antagonistic effects of IL-4. The higher IL-4 production inhibited the protective role of IFN-γ in the prenatally exposed mice. The present study showed that exposure to BPA promoted the production of T_H_2 cytokines only in adult mice, but both T_H_1 and T_H_2 cytokines in mice exposed prenatally, although percentages of CD4^+^CD25^+^ cells decreased in either case. A possible explanation is that BPA might directly act on T_H_2 cells to promote cytokine production in adult mice as demonstrated *in vitro*. This together with the decrease in Treg cells promoted the production of T_H_2 cytokines alone. In contrast, not enough BPA existed in prenatally exposed mice to promote the production of T_H_ 2 cytokines at the time of infection. Therefore, the decrease in Treg cells resulted in the promotion of T_H_1 and T_H_2 cytokine production. Further study is necessary to clarify this mechanism.

In recent years, attention has focused on the low-dose effects of EDCs. Xenoestrogens even at low levels were reported to exert estrogenic activity to affect the endocrine system (vom Saal and Hughes 2005). Our results showed that BPA at 2.5 and 5 μmol promoted T_H_ 2 cytokine production and decreased the percentages of CD4^+^CD25^+^ cells in adult mice. Similar effects were induced in offspring of dams with significantly lower doses of BPA, showing that the immune system in developing mice is affected by lower doses of BPA than that in adult mice. Prenatal exposure to EDCs in laboratory animals may cause more severe effects on the immune system than exposure during adult life. It is important to note that BPA exerted its effects at a dose of 10 nM (equivalent to 0.3 μg/kg bw/day) after prenatal administration, which is 100 times lower than 50 μg/kg bw/day (a permissible dose of BPA authorized by the U.S. Food and Drug Administration). The present results indicate that EDCs at concentrations even below the safety limit might affect our immune system.

The precise mechanism underlying the immunomodulatory effects of EDCs, especially BPA, has not been clarified, and multiple mechanisms are considered. In this study, we made a potentially important discovery, that the alteration of cytokine production induced by BPA might be mediated through a decrease in numbers of CD4^+^CD25^+^ Treg cells. Treg cells, which constitute 5–10% of peripheral CD4^+^ T cells in normal rodents and humans, are known to regulate immune responses. We found that exposure to BPA resulted in decreased percentages of CD4^+^CD25^+^ Treg cells in a dose-dependent manner in both adult and offspring mice. Because CD4^+^CD25^+^ Treg cells play a negative role in proliferation and the production of cytokines in T_H_1 and T_H_2 cells (Xu et al. 2003), decreasing numbers of CD4^+^CD25^+^ Treg cells might result in the activation of T cells. As a result, BPA exerted a stimulatory effect on the production of cytokines by the activated T cells. The decrease of CD4^+^CD25^+^ cells before infection did not result in the increased production of IL-4 and other cytokines in C57BL/6 mice. The course of infection also was not changed. This observation agrees with the result that depletion of CD4^+^CD25^+^ cells in C57BL/6 mice before infection with *L. major* did not alter the course of infection (Aseffa et al. 2002). After infection with *L. major*, the difference in percentage of CD4^+^CD25^+^ cells induced by BPA was more notable between BPA-exposed and nonexposed groups in BALB/c mice, but not in C57BL/6 mice. IL-4 may play a role in this process. The generation of peripheral CD4^+^CD25^+^ cells was induced by IL-4 (Pace et al. 2005; Skapenko et al. 2005). IL-4 production is low and only transient in C57BL/6 mice but dominant in BALB/c mice. Therefore, peripheral CD4^+^CD25^+^ cells might be decreased solely in BALB/c mice by exposure to BPA.

In conclusion, our results clearly demonstrate that the production of T_H_2 cytokines is promoted by BPA in adult mice and in offspring during developmental exposure. This suggests the possibility that BPA might cause allergy and asthma. Epidemiologic studies have shown that allergic diseases have markedly increased over the last several decades. Environmental factors such as pollutants and food additives are suspected of playing an important role. Several environmental pollutants have been reported to increase allergic responses (Bommel et al. 2000; Kato et al. 2004). BPA and other EDCs may have similar effects on allergic diseases. Furthermore, the enhanced cytokine production reported in this article was mediated through a decrease in the number of CD4^+^CD25^+^ Treg cells. Human naturally occurring Treg cells are prominent in young adults and decrease with age (Valmori et al. 2005). The decrease of Treg cells would predispose to immune dysfunction in aged individuals, explaining their higher risk of immune-mediated diseases, cancer, and infections. Our data also suggest the possibility that BPA might cause these diseases. Thus, avoiding exposure to or promoting the excretion of BPA and other EDCs would help in preventing diseases and adverse health effects.
